# Patient Preferences and Experiences in Hyperemesis Gravidarum Treatment: A Qualitative Study

**DOI:** 10.1155/2018/5378502

**Published:** 2018-10-30

**Authors:** Relin van Vliet, Marieke Bink, Julian Polman, Amaran Suntharan, Iris Grooten, Sandra E. Zwolsman, Tessa J. Roseboom, Rebecca C. Painter

**Affiliations:** ^1^University Medical Centers Amsterdam, University of Amsterdam, Amsterdam, Netherlands; ^2^Department of Gynaecology and Obstetrics, Noordwest Ziekenhuisgroep, Alkmaar, Netherlands; ^3^Department of Obstetrics and Gynaecology, University Medical Centers Amsterdam, Amsterdam, Netherlands; ^4^Department of Obstetrics and Gynaecology, University Medical Centers Amsterdam, University of Amsterdam, Amsterdam, Netherlands; ^5^Departments of Obstetrics and Gynaecology, Public Health & Epidemiology, University Medical Centers Amsterdam, University of Amsterdam, Amsterdam, Netherlands

## Abstract

**Introduction:**

Hyperemesis gravidarum (HG) medical therapies are currently of limited effect, which creates a larger role for patient preferences in the way HG care is arranged. This is the first study using in-depth interviews to investigate patients' preferences and experiences of HG treatment.

**Materials and Methods:**

We conducted individual in-depth interviews among women who had been hospitalized for HG in North Holland at least once in the past 4 years. We asked them about their experiences, preferences, and suggestions for improvement regarding the HG treatment they received. The sample size was determined by reaching data saturation. Themes were identified from analysis of the interview transcripts.

**Results and Discussion:**

13 women were interviewed. Interviewees emphasized the importance of early recognition of the severity of HG, increasing caregivers' knowledge on HG, early medical intervention, and nasogastric tube feeding. They valued a single room in hospital, discussion of treatment options, more possibilities of home-treatment, psychological support during HG and after childbirth, and more uniform information and policies regarding HG treatment.

**Conclusion:**

Further research is needed to establish whether the suggestions can lead to more (cost) effective care and improve the course of HG and outcomes for HG patients and their children.

## 1. Introduction

Nausea and vomiting of pregnancy (NVP) are common during the first trimester of pregnancy, affecting 50 to 80% of pregnant women. A much smaller proportion (0.3-3%) of pregnant women encounter intractable vomiting, which may be complicated by dehydration, significant weight loss, and electrolyte disturbances necessitating hospital admission [1]. This condition is called hyperemesis gravidarum (HG). HG has a major effect on patients' quality of life and is associated with adverse perinatal outcomes, including low birth weight, small for gestational age, and prematurity [2].

Despite the recent introduction of national HG guidelines in the UK [3], Canada [4], and USA [5], such guidance is lacking in many countries and there is considerable variation in treatment between the different hospitals in Netherlands.

Antiemetic and other HG therapies are of limited effect, which creates a larger role for patient preferences in the way HG care is arranged [6]. Few qualitative studies and patient satisfaction surveys concerning HG have been carried out. In 2000, Munch [7] found that HG patients considered the perception of being believed and taken seriously as important qualities of doctors. Power et al. [8] found that HG patients felt unpopular with caregivers. Caregivers indicated having doubts about the severity of the symptoms and the necessity for hospital admission. Taken together, the existing literature leaves health care professionals largely uninformed about what HG patients themselves would consider to constitute good HG care.

The aim of our study was to describe women's experiences and preferences of HG care. Which aspects of care did they find helpful and led to recovery? Which areas of care need improvement? What were their main concerns and needs during and after their HG episode? The findings of our study will contribute to the improvement of HG treatment as they could be used as a basis for innovation and relocation of care in such way that it better meets the needs and preferences of women with HG. Finally, the input of patients can guide the HG research agenda. 

## 2. Materials and Methods

### 2.1. Samples Selection and Recruitment of Participants

We recruited participants by posting an invitation on Facebook and home page of ZEHG, the Dutch HG patient foundation (see supplementary information 1 for the original Dutch invitation with English translation below). Patients who were interested in participating were invited to contact the researchers by e-mail. Participants were eligible for inclusion if they had been admitted to hospital for HG treatment at least once in the past 4 years in the province of North Holland. The sample size was determined by the point at which data saturation was reached. Data saturation occurs when new interviews do not provide any new data.

Verbal consent to use the voice recorder was requested at the start of the interview. All participants gave written consent for recording and using the interview as material for medical scientific research. The Medical Ethics Committee (MEC) of the Academic Medical Center decided that this study was not subject to the Medical Research Involving Human Subjects Act (WMO).

### 2.2. Data Collection

We collected data by using open and extended querying during in-depth interviews in order to provide sufficient space for asking supplementary questions. The interview started with the question “What were your experiences with the treatment you received for HG?”. When the answer to this question was too brief, supplementary questions were asked based on the topic list (see supplementary information 2). The topic list was updated continuously with the frequently named and emphasized topics from previous interviews.

During the interviews, baseline data of the women interviewed regarding their hyperemesis gravidarum experiences were obtained (see Table 1). All participants had been admitted to hospital for HG treatment in the past 4 years (inclusion criteria). We did not collect data on the time (years and months) between the interview and the HG pregnancy.

Two investigators (MB and RV) coconducted the 13 interviews, which lasted on average 40 minutes each. To minimize variation between interviews, one investigator (MB) always acted as interviewer. The second investigator functioned as observer (RV). The interviewer (MB) is an experienced midwife. The observer made a summary for the subject review and checked by using the topic list whether all topics had been addressed. Participants were given the choice of being interviewed at their homes or at the hospital.

In order to check the accuracy of the findings, member checking and subject review were performed. We carried out subject review by providing the patient with a summary of the interview in order to check the information for accuracy and correctness of interpretation [9].

### 2.3. Data Safety and Anonymity

The interviews were recorded using a recording device. With the help of two other investigators (AS and JP), the records were transcribed and transferred to a hard disk and deleted from the carrier. We ensured participant anonymity by removing the personal details from the records and transcripts. All data (interview records, transcripts, and participant's data) were labeled with numbers following the timeline of the interviews.

### 2.4. Data Analysis

All interviews were fully transcribed and coded using the three different types of coding methods of Strauss and Corbin, as described by Boeije (2008) [10]. Using open, axial, and selective coding, the most relevant and most frequently arising topics (themes) were determined and ordered. These are described in Results. The coding was done by a researcher (the observer) and checked by a second one (the interviewer). Discrepancies in the codes were resolved by discussion.

Data analysis was facilitated by MAXQDA qualitative data analysis software version 12 (VERBI Software (2016), Berlin, Germany).

## 3. Results

13 women were interviewed. Baseline characteristics of the women interviewed regarding their hyperemesis gravidarum experiences are shown in Table 1. The themes of the interviews were the attitude of caregivers towards the patient, medical treatment, psychological support, aftercare, and the information provided.

### 3.1. Theme 1: Caregivers' Attitudes

All participants stressed the importance of understanding and recognition of the disease in HG treatment. Many reported that they had not been taken seriously by caregivers, and their problems were being trivialized.

“I couldn't even keep a mouthful of water down. If a GP then still says: ‘most pregnant women feel sick, with nausea and vomiting'. I thought: ‘yes, 3 times in the morning or 30 times, the whole day long vomiting, and not being able to leave the toilet, because you can't even get on your feet any more, I think that's quite different'.”

-  *participant*  9

Also, participants perceived that caregivers thought it was their own choice not to eat and felt pressured by them.

“In hospital they said ‘you have to eat', ‘you have to get that food inside you', ‘here, this is what you get, and you have to eat it'.” But if I can't eat it because I immediately need to throw up, then I really can't eat it. If I take two bites, three come out. It's easier said than done. It isn't easy to deal with being pressured like that.”

I: How could they have done better?

R: Maybe by being kinder, like ‘try to eat, but if it doesn't work, it's no problem.' There was so much pressure.”

-  *participant*  3

According to the participants, more knowledge on HG among caregivers would have contributed to more understanding and knowledge of the disease and early recognition of the symptoms.

“The lack of knowledge on HG surprised me. Among professionals too, they think it's all over after 12 weeks.”

-  *participant*  10

### 3.2. Theme 2: Medical Treatment

#### 3.2.1. Early Medical Intervention

All participants underlined the importance of early medical intervention. They encountered that their calls for help were not taken seriously. They wanted the caregivers to explore the severity of the disease by thorough questioning and paying a home visit, because they experienced difficulties with visiting a GP or midwife.

“At one point I called and said ‘I can't manage any more, I can't even walk to the toilet without fainting'. They said ‘alright, then I want you to come to our practice'. So I said ‘how will I get there?' (…) Driving by car with HG is a nightmare. I was vomiting all the way to the practice, and in the waiting room too, with people all around me.”

-  *participant*  9

In addition, some participants had trouble with being assertive due to their sickness and weakness.

“I was very apathetic, very strange, but normally I'm quite assertive, at least I can explain what I feel very well, but not then, not at all.”

-  *participant*  2

Often treatment only starts after dehydration sets in. Interviewees reported that they would have preferred a more prevention-focused treatment, for example, by starting earlier with (other) medication and/or nasogastric tube feeding (see also next paragraph). According to them, early treatment could avoid dehydration, further weight loss, and multiple hospital admissions.

“But if I wasn't dehydrated, they sent me home, although I knew I would be back within a few days, because it wasn't a solution. (…) They let me go for so long, that I lost so much weight and was dehydrated in such way, that it went too far. (…) Waiting till I was dehydrated, only then they were willing to take action.”

-  *participant*  9

“I think if they had started earlier with rehydration and nasogastric tube feeding, the harm would have been in any case limited; I wouldn't have lost so much weight and wouldn't have been lagging behind so much.”

-  *participant*  1

#### 3.2.2. (Early) Nasogastric Tube Feeding

8 women (62%) had received nasogastric tube feeding and underlined the benefits of it. Women who were not treated with tube feeding said they wished they had. The reasons stated were the following:

- Prevention of severe weight loss, dehydration, general weakness that comes with lack of intake, and necessity for (multiple) hospital admissions
- Ensuring enough nutritional intake for the baby
- Reducing vomiting by preventing an empty stomach

“The nasogastric tube feeding provided an 80 ml drip 24 hours a day, which provided a constant base intake. (…) So my stomach stayed quite calm, so I didn't vomit and could take in the nutrients. But in my first pregnancy I lived all those months on just two white rolls; two white rolls a day and I could drink then, at least water. But you know, I was so extremely weak after giving birth, because I hadn't taken and absorbed any nutrients. Because I received nasogastric tube feeding during my second pregnancy, I just noticed that it went so much better.”

-  *participant*  9

#### 3.2.3. Communication about the Different Treatment Options

Over half of the interviewees (7/13) reported lack of communication about the different treatment options. More communication could have given them a more positive outlook and trust in continuing their pregnancy. They also wanted to have a say in deciding on which therapy was appropriate. 5 participants felt it important to make a treatment plan with the gynecologist to feel up to another pregnancy.

“What I missed is that they didn't tell me the therapeutic options; let's say they didn't even mention the words ‘nasogastric tube feeding'. Only because I started searching for something myself, because I thought: ‘Can I do something or is it just over? I mean, you don't make the choice to remove the baby just like that.”

-  *participant*  9

Furthermore, the participants indicated big differences in HG treatment between hospitals. They noticed these differences by sharing information and experiences with peers. Those differences were mainly in the pharmacological treatment but also in the criteria for hospital admission. They advocated more uniformity.

“In one hospital Ondansetron is prescribed, and in another only *Emesafene*^*∗*^. In one hospital you can get Metoclopramide and in another you can't have it because of the harmful side-effects. Then I thought there needs to be a consistent policy.”

-  *participant*  7

^*∗*^combination of meclozine and pyridoxine.

#### 3.2.4. Single Room in Hospital

The majority of the participants (69%) emphasized the importance of a single room in hospital. The main reason was avoidance of stimuli that evoked vomiting, like light, noise, and smells, in particular food, but also body odor and perfumes. Other reasons were that they were ashamed of their vomiting; it was confronting to see other more healthy and happy pregnant women, and they wanted to be alone rather than having to talk with others.

“I noticed that light really causes an extremely intense impulse to vomit. On the other side of the room a girl was admitted, and she had the television on during half the night. That was, that came in like… I just can't explain how intense those impulses are, like noise, like food, like smells, like…that really is… that's impossible to explain.”

-  *participant*  6

#### 3.2.5. Location of Therapy

6 women preferred home treatment over hospital admission so they could stay in their own familiar environment but not without effective therapy. The conditions mentioned to make home treatment possible differed. These included nasogastric tube feeding, metoclopramide or ondansetron by infusion, and also support and care at home.

#### 3.2.6. Support after Hospital Admission during Pregnancy

Over half of the participants (62%) reported lack of support and medical attention after hospital discharge for HG. This resulted in dehydration again and often the need for hospital readmission. They indicated that this could have been prevented if good home treatment, with guidance by a coach or other health professionals, had been provided.

### 3.3. Theme 3: Psychological Support

#### 3.3.1. Psychological Aid

7 women would have wanted the offer of psychological aid. Most of the women who did receive psychological support, for example, by meetings with a medical social worker (Med. SW) or a psychologist, appreciated it and considered it helpful. Women experienced loneliness, sadness, depressive feelings, anxieties, and feelings of failure and guilt.

“Of course it's an overwhelming experience if you are so nauseous… I really had the feeling that the whole world had turned black, that I had ended up in a nightmare and that things would never be alright again.”

-  *participant*  11

“I felt guilty… I felt guilty about work, I felt guilty towards my child, I felt guilty towards my husband… and I felt angry with myself: ‘why can't I do this?' (…) Yes, it would have been very nice if I could have spoken to someone other than my direct family. It doesn't have to be solved, but it helps to talk about how you can deal with it.”

-  *participant*  10

Not only psychological support by a professional but also attention for the psychological impact of HG, for example, by nurses or other caregivers, was mentioned as very important. Questions like “how are you feeling?” and “what do you need?” were very much appreciated.

#### 3.3.2. Frequent Ultrasound Checks

5 participants considered termination of a wanted and planned pregnancy due to HG symptoms. 8 participants said that seeing their baby on the ultrasound images gave them the strength to continue their pregnancies and experienced frequent ultrasound checks as very helpful and supportive.

“Each time I saw the baby on the display, I thought ‘this is what I'm doing it for, for you'. ‘You're still alive, and I need to do this for you; that's what I owe you'. ‘I'm your mum, even though you're still so small; that's what I'm fighting for'.”

-  *participant*  3

### 3.4. Theme 4: Aftercare

We defined aftercare as care after childbirth. Both physical and psychological problems did not disappear after childbirth. 9 participants (69%) would therefore have appreciated the offer of aftercare. The following suggestions were made: a dietician to help regain a normal dietary intake, a physiotherapist to help regain strength, and psychological support to help patients deal with the violent and sometimes traumatic experiences.

“I was allowed to pull out my nasogastric tube during birth. So I did that, and after that it was finished. I think that's wrong, that there is no aftercare. I wasn't able to eat normally for the first 3-4 months, so I had serious weight loss. (…) My stomach and digestive system weren't used to anything anymore, everything reacted very intensely… Well, after 5 months I still have problems with that.”

-  *participant*  9

The majority of the women wanted to be helped with handling their negative experiences. Some of them arranged help by themselves, for example, by following EMDR (eye movement desensitization and reprocessing) therapy, an empirically validated treatment for psychological trauma, and other negative life experiences [11]. Examples of complaints were stress reactions to being ill or seeing others being ill, depressive feelings, not being able to bear seeing other pregnant women, overreacting to naive remarks about HG, and the undesirability of a subsequent pregnancy.

Participants also indicated that they would have liked a follow-up consultation with the gynecologist in order to get everything straight and deal with the hard period they have been through. They would also have liked to discuss a treatment plan for a possible next pregnancy.

### 3.5. Theme 5: Provision of Information

Many participants heard very late in the course of their illness that they were suffering from HG. According to them, it is important that HG is diagnosed and named as a separate entity from NVP and recognized earlier and that good information about the disease, the treatment options, and prognosis is provided. Women reported that the knowledge of having a “real” disease and not just morning sickness already helped them. This made them feel like they were not exaggerating, but they were suffering from a severe disease. In particular, the Facebook group and the page of ZEHG foundation (the Dutch HG patient foundation) were deemed valuable. Patients could find support, tips, and advice from peers and information about HG. They could inform colleagues, friends, and family to create understanding.

“If I had known earlier about foundation ZEHG… I found a lot of information there, and I shared that with my boyfriend, my parents and his parents, who all didn't understand… These were the people from whom I hoped to receive help, and from whom I eventually did, but only after all the information, because they just didn't understand it at first, they just couldn't imagine it.”

-  *participant*  6

Participants suggested that the provision of information for HG patients and their families could improve by providing patient information sources like ZEHG foundation, for example, by means of a flyer.

## 4. Discussion

### 4.1. Main Findings

Using unstructured interviews, we show that women who had been hospitalized for HG in North Holland in the past 4 years identified several areas of improvement in HG care, including increasing caregivers' knowledge on HG, early medical intervention and nasogastric tube feeding, a single room in hospital, discussion of treatment options, more possibilities of home-treatment, psychological support during HG and after childbirth, and more uniform information and policies regarding HG treatment.

### 4.2. Strengths and Limitations

This is the first study using in-depth interviews to investigate patients' preferences and experiences of HG treatment. However, our study has some limitations. We recruited participants by publishing a call for participants on the website and in Facebook groups of the “ZEHG” foundation. This strategy may have led to selection bias, oversampling the opinions of women with severe HG or of those who were active members of the online patient community. Furthermore, the recruitment text mentioned our aim to improve the treatment of HG. Women who were satisfied with the care they received may not have responded.

Despite possible selection bias, several items named by the participants correspond with existing literature, which will be discussed under the subheading “Interpretation.” A strength of our study was that our sample already reached saturation at 13 interviews, indicating a high degree of interpersonal consistency, although this might simply reflect that our recruitment strategy yielded a homogeneous sample.

Another limitation is the fact that the interviewees were all inhabitants of a single region and country. Netherlands lacks a national guideline for HG treatment. It is likely that variations in treatment between regions and countries have significant effects on topics that might improve HG care. In UK, for example, many women with HG may be cared for within Early Pregnancy Units [12], rehydration in day care is widely available [13], and there currently is a national guideline for HG [3]. On the other hand, in Netherlands, women with HG are likely to be first cared for by their community midwife close to home and are more likely to be seen by a dietician (personal communication) than in most other countries. And, in Norway, tube feeding is a more common therapy [14]. It is unknown what effects these (organizational) aspects have on women's experiences with HG care.

### 4.3. Interpretation

The participants underlined the need for empathy and recognition for their illness. This corresponds with existing literature. For example, the study by Munch [7] found that the perception of being believed and taken seriously by doctors was important for HG patients. Power et al. [8] found that HG patients felt unpopular with caregivers and perceived to be “time wasters.” Their perception seemed to be justified as caregivers indicated to have doubts about the severity of the symptoms and the necessity for hospital admission. Furthermore, the need of patients to receive empathy and recognition is also described by qualitative research into other diseases, like fibromyalgia [15].

Participants believed that medical intervention and nasogastric tube feeding, introduced at an early point in treatment, are helpful by avoiding weight loss, repeated admissions, and general weakness. Our participants' opinion is at odds with the findings from the first RCT into the potential benefits of early nasogastric tube feeding, which demonstrated no benefit in an unselected population of women admitted to hospital for HG [16]. As regards other early medical interventions, there is some support for the notion of a preemptive approach to HG: a small study among women with a history of severe NVP and HG demonstrated significantly less severe symptoms in the subsequent pregnancy among women who had been randomly assigned to preemptive Diclectin compared to those assigned to commencing medication after symptoms developed [17, 18].

Participants indicated that certain stimuli, in particular smells, provoked vomiting and aggravated their disease. Olfaction is indeed thought to be a strong trigger for NVP symptoms [19, 20]. A single room could lead to a reduction of these stimuli and increase well-being. Participants would have favored home-treatment over hospital admission but only if the necessary facilities and home assistance were in place. Unfortunately, there is little known about the effect of single room and home-treatment on the course of HG. McCarthy et al. did describe that, in comparison to inpatient management, day care management is less costly and equally acceptable [13]. Based on our findings, future studies could investigate (cost) effectiveness and satisfaction for expanded home-care options for HG.

HG had a large psychological impact on our participants. According to them, psychological support, during pregnancy and afterwards, is essential. Previous studies also described this psychological impact: McCormack et al. [21] found women with HG to be at elevated risk of mental health difficulties during pregnancy, Christodoulou-Smith et al. [22] found that 18% of women following HG pregnancies reported full criteria PTSS, and Mazotta et al. [23] found consideration or actual termination of pregnancy due to NVP to be associated with depressed feelings. However, little published evidence on the effects of psychological intervention for HG patients is available. The RCT of Faramarzi et al. [24] concluded that psychotherapy added to medical therapy yielded significant improvements in NVP-specific and anxiety/depression symptoms, compared with medical therapy alone among women with NVP. Further research into the efficacy of psychological interventions for HG patients is required.

Physical support after childbirth, for example, by a dietician and/or physiotherapist was also valued. Again, these aspects of care have not been assessed in clinical trials. Furthermore, participants suggested providing uniform information about the course of HG in leaflet form to all patients, managing expectations of family, friends, and colleagues. Finally, patients' call for more uniformity could be achieved by a national guideline. At the time of our survey, Netherlands lacked one. Three factors may have contributed to this. First, until recently, there was lack of aggregated evidence on the efficacy of various treatment options for HG. The Cochrane review [6] and systematic review of McParlin et al. [25] both concluded that there is little high-quality evidence supporting any intervention for HG treatment and highlighted the need for more high-quality trials and a uniform definition and core outcome set for HG [6, 26]. However, they did emphasize that some antiemetic medication is effective for treating HG. Second, possibly as a result of the previous lack of aggregated evidence, only in the past 3 years UK, Canada, and USA have issued practice guidelines for HG. Finally, both the lack of curative options and the low prevalence mean HG only has received limited attention in medical training schemes. Taken together, the recent developments, that is, publication of aggregated evidence and national guidelines, may improve uniformity in treatment as well as the knowledge on HG among medical professionals.

## 5. Conclusions

The purpose of this study was to explore the experiences and preferences of HG treatment of women suffering from HG over the past 4 years in order to improve HG care. Patients stressed the need for more knowledge among caregivers and early recognition and medical intervention. Also, a number of organizational aspects including admission in a single room, home-care options, and more support after admission were mentioned. Further research needs to be done to establish whether these suggestions can indeed lead to more (cost) effective care and could improve the course of HG as well as outcomes for HG patients and their children.

## Figures and Tables

**Table 1:** Baseline data of the women interviewed regarding their hyperemesis gravidarum experiences.

	**Age**	**G+P**	**Treatment**	**Psychological support during HG**	**After-care**	**Involved health givers**
1	30	G4P0	Meclozine, Metoclopramide, rehydration, nasogastric tube feeding, total parenteral nutrition	Med. SW	EMDR	GP, psychiatrist, neurologist, gynaecologist, midwife, dietician, Med. SW, psychologist

2	27	G1P0	Meclozine, Metoclopramide, rehydration		EMDR	GP, gynaecologist midwife psychologist

3	28	G1P0	Meclozine, Metoclopramide Ondansetron, rehydration, nasogastric tube feeding		Meetings with a psychologist	GP, midwife, gynaecologist psychologist

4	27	G1P0	Metoclopramide, Nutridrink, rehydration, nasogastric tube feeding	Med. SW, meeting with a psychologist	Meetings with a psychologist	GP, midwife, gynaecologist, Med. SW psychologist

5	21	G1P0	Medication unknown, rehydration	Med. SW	Meetings with a psychologist	GP, gynaecologist psychologist

6	30	G1P0	Meclozine, rehydration nasogastric tube feeding	Med. SW		GP, midwife gynaecologist Med. SW dietician

7	19	G3P0	Meclozine, Metoclopramide, rehydration	Meetings with a psychologist	EMDR in future	GP, gynaecologist psychologist

8	28	G1P0	Meclozine, Nutridrink, rehydration	Meetings with a psychologist	EMDR	GP, midwife, gynaecologist psychologist

9	30	G2P1	Meclozine, Metoclopramide, Ondansetron, rehydration nasogastric tube feeding	Med. SW	EMDR, dietician	GP, midwife gynaecologist, Med. SW, psychologist, dietician

10	30	G3P2	Meclozine, Metoclopramide, rehydration, nasogastric tube feeding		Meetings with a nurse practitioner and Internist	GP, midwife gynaecologist, nurse practitioner

11	33	G2P1	Meclozine, Metoclopramide, Haldol, Ondansetron, rehydration, nasogastric tube feeding	Meetings with a psychiatrist		Midwife, gynaecologist, psychiatrist

12	27	G2P0	Meclozine, Nutridrink, rehydration			GP, midwife gynaecologist dietician

13	31	G2P1	Meclozine, Ondansetron, rehydration	Med. SW, meetings with a psychologist		GP, midwife gynaecologist, Med. SW, psychologist

*G: gravidity, P: parity, Med. SW: medical social work, EMDR: eye movement desensitization and reprocessing*, *GP: general practitioner.*

## Supplementary Materials

Supplementary 1 Supplementary information file 1: “Call for participation in research for HG treatment”: our call for participation posted on Facebook and home page of ZEHG, the Dutch HG patient foundation. The original document is written in Dutch followed by English translation below.

Supplementary 2 Supplementary information 2: “Topiclist”: the interviews started with the question “What were your experiences with the treatment you received for HG?”. Supplementary questions were asked based on this topic list and the observer used them to check whether all topics had been addressed. The topic list was updated continuously with the frequently named and emphasized topics from previous interviews.
