# Supplementary material for: Patient Preferences and Experiences in Hyperemesis Gravidarum Treatment: A Qualitative Study
Source: J Pregnancy. 2018 Oct 30;2018:5378502. doi: 10.1155/2018/5378502 (PMC6234451; doi:10.1155/2018/5378502)
Supplement: Supplementary 1 — Supplementary information file 1: “Call for participation in research for HG treatment”: our call for participation posted on Facebook and home page of ZEHG, the Dutch HG patient foundation. The original document is written in Dutch followed by English translation below. [file 5378502.f1.docx]

**Supplementary information 1**

*English translation below*

We ontvingen de volgende oproep om deel te nemen aan onderzoek naar de behandeling van hyperemesis gravidarum. Heb je HG gehad en ben je opgenomen geweest in een ziekenhuis in Noord Holland? Dan kun je deelnemen aan het onderzoek! En daarmee kun je een bijdrage leveren aan het verbeteren van de zorg rondom HG.

Waarom dit onderzoek?

Elk jaar weer is hyperemesis gravidarum één van de meest voorkomende redenen voor een ziekenhuisopname bij zwangere vrouwen. De zorg die vrouwen dan krijgen verschilt echter van ziekenhuis tot ziekenhuis. Vanuit de afdeling Gynaecologie van het AMC onderzoeken we welke zorg vrouwen ontvangen als zij door HG te maken krijgen met een ziekenhuisopname. Welke zorg werkt en waar kan deze zorg nog verbeterd worden? Om een volledig beeld te krijgen, hebben we uw hulp nodig! Wat zijn uw ervaringen met zorg die u heeft ontvangen voor een ziekenhuisopname voor HG? Waar ziet u nog kansen of verbeterpunten voor deze zorg?

Wie kan deelnemen aan dit onderzoek?

Wij zijn op zoek naar vrouwen die HG gehad hebben en in de afgelopen 4 jaar voor HG zijn behandeld met een ziekenhuisopname in een ziekenhuis in de regio Noord Holland.

Hoe ziet het onderzoek er uit?

Het onderzoek bestaat uit een interview van ongeveer 45 minuten. In het interview vragen wij naar uw ervaringen met de behandeling die u voor HG heeft ontvangen. Dit interview vindt plaats in het AMC in Amsterdam, maar kan in overleg ook op een andere locatie plaatsvinden of via Skype gedaan worden.

We verwerken uw gegevens geheel vertrouwelijk en anoniem.

Wat doen we met de interviews?

Na het afnemen van de interviews analyseren wij uw ervaringen en vergelijken we de verschillende interviews met elkaar. Zo krijgen we een goed beeld over wat er volgens u goed gaat in de zorg voor HG en wat er beter kan. Met deze informatie kan dan bijvoorbeeld worden bepaald wat de onderwerpen voor toekomstig onderzoek zouden moeten zijn! Wij zullen het verslag over ons onderzoek ook op de website van stichting ZEHG plaatsen.

Is uw interesse gewekt na het lezen over dit onderzoek? U kunt zich aanmelden door een email te sturen naar onderzoekamc@zehg.nl. Ook als u nog vragen heeft over dit onderzoek, kunt u een email sturen naar dit mailadres.

**English translated version**

Call for participation in research for HG treatment

We received the following request for participation in a study for Hyperemesis Gravidarum treatment. Have you suffered from HG and have been admitted to a hospital in North Holland? If so, you can participate in the study! Doing so, you are able to contribute to improving healthcare concerning HG.

**REQUEST**

Why this study?

Every year, Hyperemesis Gravidarum appears to be one of the main causes of hospital admission for pregnant women. The healthcare that these women receive, however, dramatically differs between hospitals. The department of gynaecology of the AMC wants to study what kind of treatment women with HG get when they are admitted to the hospital due to HG. Which types of therapy work and where can we make improvements? In order to get a good view of the situation, we need your help! What are your experiences with the healthcare you have received during your hospital admission for HG? What opportunities for improvement do you see?

Who can participate in the research?

We are looking for women who have suffered from HG and have been treated in the last four years for HG by hospital admission in the area of North Holland.

What does the study look like?

The study concerns an interview (approx. 45 min). In this interview, we will ask about your experiences with HG treatment. This interview will be conducted in the AMC in Amsterdam, but can also be relocated elsewhere or be conducted via Skype.

We will process your data confidentially and anonymously.

What will we do with the interviews?

After the interview is conducted, we will analyze your experiences and compare the different interviews. By doing this, we will provide a good overview of what is going well in care for HG and what can be improved. With this information we can determine what should be the subjects for future research! We will post our report on the study on the website of the ZEGH foundation.

Did we interest you in participating after reading this? You can submit by sending an email to onderzoekamc@zegh.nl. May you have further questions concerning the study, please send your questions to the email address mentioned above.
