# Supplementary material for: Patient Preferences and Experiences in Hyperemesis Gravidarum Treatment: A Qualitative Study
Source: J Pregnancy. 2018 Oct 30;2018:5378502. doi: 10.1155/2018/5378502 (PMC6234451; doi:10.1155/2018/5378502)
Supplement: Supplementary 2 — Supplementary information 2: “Topiclist”: the interviews started with the question “What were your experiences with the treatment you received for HG?”. Supplementary questions were asked based on this topic list and the observer used them to check whether all topics had been addressed. The topic list was updated continuously with the frequently named and emphasized topics from previous interviews. [file 5378502.f2.docx]

**Supplementary information 2**

Topiclist

- What kind of healthcare have you received and from whom?
- How did you experience your treatment/care during your pregnancy with heavy nausea and vomiting (HG)?
- What were the positive and negative aspects of the different forms of treatment?
- How did you experience the care you received by the different health practitioners? (hospital, general practitioner, gynecologist, midwife, dietarian)
- What were the most relevant worries and did those worries receive sufficient attention?
- Did you receive psychological support? If so, has it been helpful?
- What were the most relevant needs/wishes for you and what is the extent to which those were fulfilled?
- What suggestions do you have for improving the treatment for HG?
